# Locked compression plating versus retrograde intramedullary nailing in the treatment of periprosthetic supracondylar knee fractures: a systematic review and meta-analysis

**DOI:** 10.1186/s13018-021-02222-x

**Published:** 2021-01-22

**Authors:** Henry Magill, Nikhil Ponugoti, Amr Selim, James Platt

**Affiliations:** 1 Orthopaedic Registrar, Chelsea and Westminster Hospital, London, UK; 2 Orthopaedic Registrar, Basingstoke and North Hampshire Hospital, Basingstoke, UK; 3 Orthopaedic Registrar, Royal Cornwall Hospital, Truro, UK; 4 Consultant Trauma & Orthopaedic Surgeon, Hillingdon Hospital, London, UK

**Keywords:** Trauma, Fracture, Fixation, Periprosthetic, TKR

## Abstract

**Background:**

Periprosthetic fractures of the distal femur above a total knee arthroplasty (TKA) have traditionally been managed by locking compression plating (LCP). This technique is technically demanding and is associated with high rates of non-union and revision. More recently, retrograde intramedullary nailing (RIMN) has been proposed as an acceptable alternative. This meta-analysis aims to evaluate clinical outcomes in patients with periprosthetic supracondylar femoral fractures who were treated with LCP and RIMN.

**Methods:**

An up-to-date literature search was carried out using the pre-defined search strategy. All studies that met the inclusion criteria were assessed for methodological quality with the Cochrane’s collaboration tool. Operative time, functional score, time-to-union, non-union rates and revision rates were all considered.

**Conclusion:**

Ten studies with a total of 531 periprosthetic fractures were included. This meta-analysis has suggested that there is no significant difference in any of the outcome measures assessed. Further, more extensive literature is required on the subject to draw more robust conclusions.

## Background

Periprosthetic supracondylar fractures (PSF) of the distal femur are classified as fractures within 15 cm of the total knee replacement (TKR) [1]. These fractures are relatively rare; they occur in 0.25–2.3% of primary TKRs and significantly higher in revision cases [2–5]. The incidence is expected to increase further due to growing population longevity and provision of service [2, 4, 6].

The treatment of these fractures remains a challenge for orthopaedic surgeons. Historically, the literature reports relatively high complication rates such as non-union and revision in the setting of traditional non-locking plate fixation [7, 8]. Outcomes are complicated by the fracture configuration, fracture stability, implant type, metaphyseal bone quality and surgical experience [9, 10].

More recently, the use of both locking compression plates (LCP) and retrograde intramedullary nailing (RIMN) has proved successful methods of fixation [7, 11, 12]. However, the ideal treatment modality remains controversial where a consensus is not clear in the literature [7, 12–20].

The aim of this study is to examine the clinical outcomes for LCP and RIMN in the treatment of periprosthetic femur fractures around a TKR and provide the most up-to-date, level I evidence in the literature. We will specifically compare operative time, knee function, time-to-union, non-union rate and revision rate between the two groups.

## Methods

### Literature search

The systematic reviews and qualitative analysis were performed in accordance with the Preferred Reporting items for Systematic reviews and Meta-analyses (PRISMA) [21] and the Cochrane Handbook for Systematic Review of Intervention [22]. We have searched Medline, Embase and Cochrane library databases from Jan 2000 to May 2020. All case reports and narrative reviews were excluded. The search was performed on the following areas: ‘Periprosthetic fracture’ [Mesh] and Distal femur or ‘knee arthroplasty’ [Mesh] and ‘locking plate’ [Mesh], or ‘intramedullary nail’ [Mesh].

### Searching other resources

A further search was performed for any other previously published, planned and on-going trials by identifying references in ClinicalTrials.gov (http://clinicaltrials.gov/) and the World Health Organization (WHO) International Clinical Trials Registry (http://apps.who.int/trialsearch/).

### Inclusion and exclusion criteria

All search terms, titles, abstracts and full text of articles that were deemed suitable for abstract were reviewed.

Inclusion criteria are as follows:

1. Level I, level II, and level III (comparative studies) evidence;
2. Studies directly comparing locking plate versus retrograde intramedullary nail for treating periprosthetic supracondylar fractures.
3. Skeletally mature patients (older than 18 years);
4. All original research and comparative studies where at least one of the selected functional outcomes is reported;
5. Follow-up of at least 6 months;
6. English language only.

Exclusion criteria are as follows:

1. Cadaveric or animal studies;
2. Case reports, letters, abstracts and conference articles;
3. Repeated studies and data;
4. Studies that did not meet the above criteria.

### Outcome measures

The primary outcome measures of interest for this review were as follows:

1. Operative time (OT);
2. Knee function score (KSS/WS/OKS);
3. Time to union (TTU);
4. Non-union rate (NUS);
5. Revision rate (RR).

### Data extraction

Data were extracted separately by two reviewers; the basic demographics for each study were first author, year of publication, study design, sample size, mean age and the outcome parameters measured. Any disagreements were solved through discussion between the two reviewers, and in the case of conflict, a further reviewer is consulted.

### Data synthesis and statistical analysis

Mean difference was used to analyse continuous variables whereas risk difference was used to analyse dichotomous variables. Review Manager 5.3 was used for all data synthesis and analysis. *P* < 0.05 was considered statistically significant with confidence intervals (CI) set to 95%. The ‘random effects model’ was applied if significant heterogeneity existed between the compared studies. Final results for each parameter were displayed in a forest plot. Chi square test was used to analyse heterogeneity between the studies, and heterogeneity size was formally determined with *I*^2^ (where 0–25% indicates low heterogeneity, 25–75% indicates moderate heterogeneity, and > 75% suggests high heterogeneity).

### Methodological quality assessment

All the studies included in this meta-analysis were retrospective cohort studies. All studies were formally assessed for quality according to the Newcastle Ottawa scale [23]. This scale uses a star system ranging from 0 to 9. High quality studies were those who scored more than six stars.

## Results

### Literature search results

**Fig. 1:**
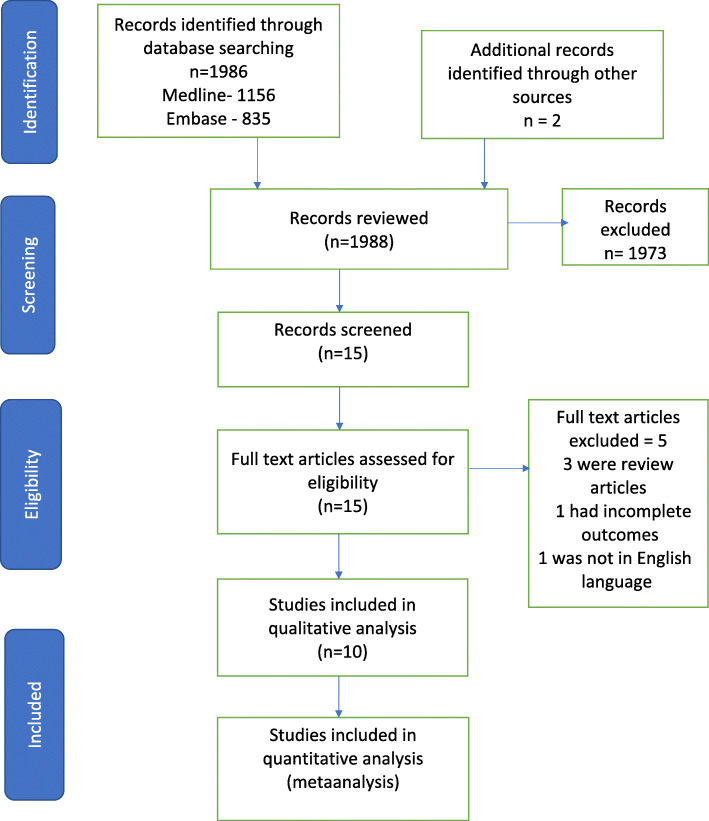
The Preferred Reporting Items for Systematic reviews and Meta-analysis

The initial search of the Medline and Embase databases resulted in 1986 articles, and 2 other articles were added to literature search from other sources. Titles and abstracts of the articles were reviewed, and 15 articles were deemed eligible for screening. Out of 15 articles, 5 studies were excluded, as they do not meet the inclusion criteria. Finally, 10 cohort studies were included in the meta-analysis for qualitative and quantitative assessment. The PRISMA flowchart is shown in Fig. 1.

### Quality assessment

The non-randomised studies were assessed for quality using the Newcastle Ottawa score with a subjective score out of 9. All the included studies are of good quality with 2 or 3 stars in the selection domain, 1 or 2 in comparability domain and 2 or 3 in outcome/exposure domain. All scores are displayed in Table 1

**Table 1:** The methodological index for non-randomised studies (Newcastle Ottawa Scale)

References	Selection	Comparability	Outcome	Total score
**Aldrian et al.** [12]	3	2	3	8
**Gondalia et al.** [11]	2	2	3	7
**Horneff et al.** [9]	3	2	3	8
**Hou et al.** [24]	3	1	3	7
**Kilucoglu et al.** [25]	2	2	3	7
**Kyriakidis et al.** [26]	3	2	3	8
**Large et al.** [19]	2	2	3	7
**Meneghini et al**. [10]	3	2	2	7
**Matlovich et al.** [27]	2	2	3	7
**Park et al.** [28]	2	2	3	7

### Characteristics of studies included

The 10 cohort studies included in the study are reviewed in Table 2. All included studies were published between 2008 and 2019. A total of 531 periprosthetic fracture cases were included in the study, of which 320 were treated with LCP and 211 had RIMN. The follow-up time of the involved studies ranged from 8.4 (mean) to 51.6 months (mean).

**Table 2:** Characteristics of included studies

Author	Year	Study type	Sample size		Age (mean, years)		Measured parameters	Period of follow-up	Fracture classification	Operative time		Knee society scores		Time to union		Non union		Revision surgeries	
			LCP	RIMN	LCP	RIMN				LCP	RIMN	LCP	RIMN	LCP	RIMN	LCP	RIMN	LCP	RIMN
Aldrian et al. [12]	2013	RCS	48	36	75.6	75.6	NUR, RR	At least 12 months	Su types I, II, III							7 (48)	3 (36)	10 (48)	6 (36)
Gondalia et al. [11]	2014	RCS	24	18	69.9	69.9	OT, KSS, TTU, NUR, RR	No description	AO 33 A1, A2, A3	135 ± 31.9 (24)	125 ± 38.5 (18)	76.5 ± 14.5 (24)	80.6 ± 10.9 (18)	49.8 ± 42.5 (24)	38.3±25.5 (18)	7 (24)	3 (18)	7 (24)	5 (18)
Horneff et al. [9]	2013	RCS	28	35	68.3	69.5	OT, TTU, NUR, RR	At least 36 months	L&R type II	155 ± 50 (28)	113 ± 35 (35)			11.5 ± 6 (28)	12.4 ± 7 (35)	0 (28)	8 (35)	4 (28)	14 (35)
Hou et al. [24]	2012	RCS	34	18	75	77	OT, TTU, NUR	Mean 28.8 months	OTA	87 ± 6.4 (34)	92 ± 6.8 (1)			16 ± 4.08 (34)	14.8 ± 4.2 (18)	3 (34)	1 (18)		
Kilucoglu et al. [25]	2013	RCS	9	7	76.7	69	KSS, TTU	Mean 51.6 months	Neer types II and III			78.8 ± 6 (9)	72.7 ± 8 (7)	15.6 ± 3.6 (9)	15.4 ± 2.8 (7)				
Kyriakidis et al. [26]	2019	RCS	31	29	76.1	82.1	OKS, TTU, NUR	Mean 19.7 months	L&R types I, II	64.16 ± 16.87	64.64 ± 19.37	30.8 ± 8.1 (31)	31.03 ± 9.3 (29)	5 (4–9)	6 (4.5–6)	0 (31)	1 (29)		
Large et al. [19]	2008	RCS	24	7	74.8	74.8	NUR	Mean 25.2 months	L&R type II							0 (24)	2 (7)		
Matlovich et al. [27]	2017	RCS	38	19	75.7	75.4	KSS, TTU, NUR, RR	At least 12 months	L&R type II			35.8 ± 29.22 (38)	25.9 ± 25.1 (19)	5.11 ± 4.2 (36)	3.7 ± 3.6 (19)				
Meneghini et al. [10]	2014	RCS	63	22	74	74	NUR	Mean 8.4 months	L&R type II							7 (63)	2 (22)		
Park et al. [28]	2016	RCS	21	20	75	73.9	WS, TTU, NUR	At least 12 months	L&R types I, II			24.3 ± 10.49 (21)	27.4 ± 7.85 (20)	15.29 ± 2.13	18.25 ± 8.25	0 (21)	0 (20)		

### Outcome 1: operative time

**Fig. 2:**
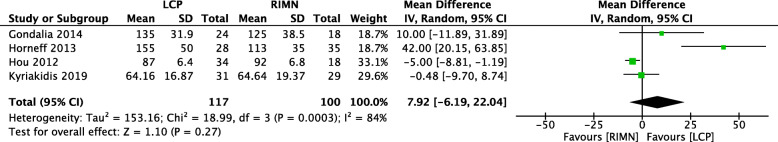
A forest plot showing the comparison of operative time (min) between the two fixation methods. CI, confidence interval; IV, independent variable; M-H, Mantel-Haenszel; LCP, locking compression plate; RIMN, retrograde intramedullary nail

The operative time was reported in 4 studies with a high level of heterogeneity (*I*^2^ = 84%) [9, 11, 24, 26]. The difference between the LCP and RIMN groups in terms of operative time was not statistically significant (Fig. 2).

### Outcome 2: knee functional score

**Fig. 3:**
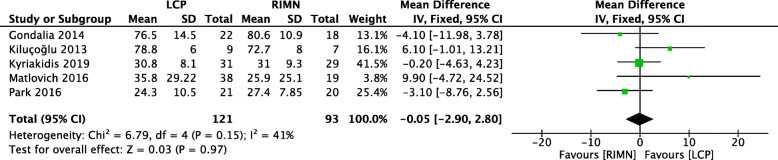
A forest plot showing the comparison of knee functional scores between the two fixation methods. CI, confidence interval; IV, independent variable; M-H, Mantel-Haenszel; LCP, locking compression plate; RIMN, retrograde intramedullary nail

Knee functional scores were reported in 5 studies with a moderate level of heterogeneity (*I*^2^ = 41%) [11, 25–28]. The comparative analysis suggests no significant difference between the LCP and RIMN groups (Fig. 3).

### Outcome 3: time to union

**Fig. 4:**
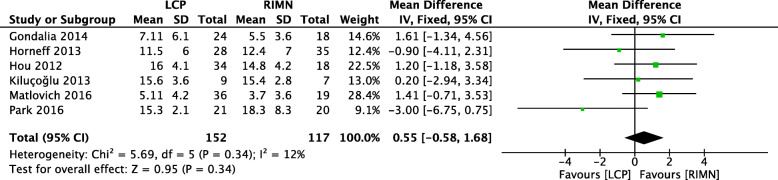
A forest plot showing the comparison of time to union (months) between the two fixation methods. CI, confidence interval; IV, independent variable; M-H, Mantel-Haenszel; LCP, locking compression plate; RIMN, retrograde intramedullary nail

The time to union was reported in 6 studies (*n* = 117) with a low level of heterogeneity (*I*^2^ = 12%) [9, 11, 24, 25, 27, 28]. Results from Kyriakidis et al.’s study were not included in the forest plot because the mean and standard deviation could not be derived from the reported data [26]. The difference between the LCP and RIMN groups in terms of time-to-union was not statistically significant (Fig. 4).

### Outcome 4: non-union rate

**Fig. 5:**
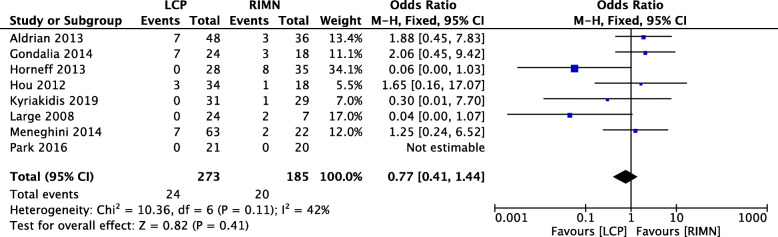
A forest plot showing the comparison of non-union rate between the two fixation methods. CI, confidence interval; IV, independent variable; M-H, Mantel-Haenszel; LCP, locking compression plate; RIMN, retrograde intramedullary nail

The non-union rate was reported in 8 studies (*n* = 185) with a moderate level of heterogeneity (*I*^2^ = 42%) [9–12, 19, 24, 26, 28]. The comparative analysis suggests that no significant difference exists between the LCP and RIMN groups in terms of non-union rate (Fig. 5).

### Outcome 5: revision rate

**Fig. 6:**
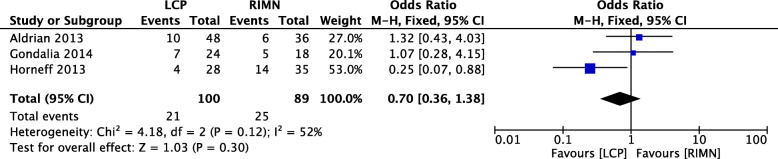
A forest plot showing the comparison of revision rate between the two fixation methods. CI, confidence interval; IV, independent variable; M-H, Mantel-Haenszel; LCP, locking compression plate; RIMN, retrograde intramedullary nail

Revision rate was reported in 3 studies (*n* = 98) with a moderate level of heterogeneity (*I*^2^ = 52%) [9, 11, 12]. The difference between the LCP and RIMN groups in terms of revision rate was not statistically significant (Fig. 6).

### Sensitivity analysis

A sensitivity analysis was performed on all comparisons where both a fixed and random effects model was applied; all results remain unchanged after sensitivity analysis.

## Discussion

To our knowledge, this is the most up-to-date meta-analysis to compare locking compression plate with retrograde intra-medullary nail fixation for the treatment of periprosthetic supracondylar knee fractures. The results from the meta-analysis demonstrate that clinical outcomes, including operative time, functional score, time-to-union, non-union rates and revision rates did not differ significantly in patients who underwent LCP or RIMN fixation.

It is evident that the treatment of peri-prosthetic supracondylar femur fractures remains challenging. Many complications have been reported in the treatment of periprosthetic supracondylar fractures; the literature suggests that this is attributed to the comparatively older populations and lower levels of experience of surgeon involved [11].

The use of IMNs fixation, at large, provides good biomechanical stability with minimal soft tissue disruption with the aim of preserving local fracture biology and therefore healing potential [10, 11, 29]. In the context of a periprosthetic TKR injury, it must be noted that they can be only used with open-box design prosthesis, few modern PS designs and non-stemmed femoral components. A closed box design poses challenge to make a nail entry usually requiring drilling with a burr [30]. Fracture comminution may also preclude the use of RIMN fixation. In addition, a proximal intramedullary device or hip prostheses may also prevent the use of a RIMN.

The results from this meta-analysis do not support the theoretical advantage of RIMN over LCP. It is possible that the disruption of the periosteal blood supply is less than originally expected; this may be due to more minimally invasive techniques or the more recent LCP designs that preserve periosteal tissue [31]. Furthermore, RIMN fixation involves indirect fracture reduction, which may result in incomplete primary reduction and the potential for secondary loss of reduction [12]. Large et al. has hypothesised that RIMN may not adequately fill the metaphyseal flare and therefore allow toggling of the distal fragment [19].

Two systematic reviews suggest that LCP fixation is associated with a lower overall complication when compared to RIMN in treating PSF [32, 33]. The most commonly quoted complications for LCP were hardware failure (5%), malunion (2.5%) and deep infection (2.2%) where for RIMN, malunion (11.5%), hardware failure (6.3%) and peri-implant fracture (1.9%) were often cited. However, more recent literature, which includes a meta-analysis by Shin et al., suggests no significant difference in the complication rates between the two techniques [13, 34]. This is in keeping with the findings of our meta-analysis where there was no significant difference in non-union or reoperation rates.

We acknowledge the limited numbers involved and quality of the included data. We also appreciate that all of the papers included are retrospective cohort studies that lack randomisation and therefore may lower the quality of the data included. The operating surgeon commonly determined the fixation method and choice of implant; this is likely to be influenced by familiarity with each technique, the trend within each institution and the fracture type. We were unable to delineate any reliable data from the included studies to determine if fracture pattern or bone quality altered the choice of fixation method. Furthermore, the meta-analysis includes subjects across many different departments and surgeons of varying ability and experience due to relatively longer learning curve to perform these procedures in periprosthetic fractures.

Fluoroscopy time was not mentioned in any of the included studies. In addition, full weight-bearing status and return to activity were not included in our comparisons as only a few studies mentioned these outcomes. Successful outcomes were measured only by way of knee functional scores and time to union.

There is no clear advantage of either RIMN or LCP in terms of successful post-op rehabilitation. It is noted that most of the studies allowed ‘weight bearing as tolerated’ for both treatment groups with no record of when a successful post-operative mobility status was achieved for each treatment choice. Specifically, Large et al. followed a protocol of ROM and quadriceps strengthening from day 1, partial weight bearing at 4 weeks and full bearing in 12 weeks [19]. Interestingly, Meneghini et al. mentioned that time to weight bearing is lower in RIMN (9.1 weeks) when compared to LCP (11.7 weeks) [10]. However, the remainder of included studies has suggested no significant difference in terms of successful rehabilitation between the RIMN and LCP groups, by any measure.

Newer methods of treating peri-prosthetic distal femur fracture that have also been successful are nail-plate technique and distal femur replacement (DFR) [35, 36]. Fractures with insufficient distal bone stock, unstable implant and incompetent ligaments are not amenable to treatment with LCP or RIMN. The alternative treatment in such cases is DFR. Moreover, recent literature on DFR suggests good outcomes in elderly patients where it allows early mobilisation and weight bearing, thereby reducing hospital stay and adverse sequela of prolonged immobilisation [37, 38]. Further research on DFR is needed to provide a stronger consensus for this treatment method.

## Conclusion

The results of this meta-analysis suggest there is no significant difference between LCP and RIMN in terms of operative time, functional score, time to union, non-union rates and revision rates for the treatment of periprosthetic supracondylar knee fractures. The results of this study indicate either management option remains an acceptable choice for the treatment of periprosthetic supracondylar fractures. The authors recommend that the chosen fixation method is determined by the surgeon’s expertise, where stable fixation can be achieved. Undoubtedly, more extensive literature is required to draw more robust conclusions

## Data Availability

Not applicable
